# Colon cancer in Luxembourg: a national population-based data report, 1988–1998

**DOI:** 10.1186/1471-2407-5-52

**Published:** 2005-05-24

**Authors:** René Scheiden, Paul Pescatore, Yolande Wagener, Nelly Kieffer, Catherine Capesius

**Affiliations:** 1 Division of Anatomical Pathology, National Health Laboratory, Luxembourg; 2 Department of Gastroenterology, Clinique Sainte-Thérèse, Luxembourg; 3 Division of preventive medicine, National Health Direction, Luxembourg; 4 Morphologic Tumour Registry, Luxembourg

## Abstract

**Background:**

Over the last two decades time trends in incidence rates of colorectal cancer, changes in the proportions of stage at diagnosis and changes in the anatomic sub-site distribution of colon cancers have been reported in some European countries. In order to determine a strategy for early detection of colon cancer in the Grand-Duchy of Luxembourg, all consecutive colon adenocarcinomas diagnosed during the period 1988–1998 at a nation-wide level were reviewed.

**Methods:**

The population-based data of the national Morphologic Tumour Registry report all new high-grade adenomas (i.e. high-grade intraepithelial adenomatous neoplasias) and all consecutive new invasive adenocarcinomas of the colon diagnosed in the central department of pathology. Attention has been focused on variations in incidence, stage, anatomical site distribution and survival rates. Rectal cancers were excluded.

**Results:**

Over the study period, 254 new colonic high-grade adenomas and 1379 new invasive adenocarcinomas were found; the crude incidence rates of colon adenocarcinomas grew steadily by 30%. Comparing the two 5-year periods 1988–1992 and 1994–1998, the crude incidence rates of high-grade adenomas (stage 0) rose by 190%, that of stage I cases by 14.3%, stage II cases 12.9% and stage III cases 38.5%, whereas the crude incidence rates of stage IV cases decreased by 11.8%. The high-grade adenoma/adenocarcinoma ratio increased. The right-sided colonic adenocarcinomas in elderly patients (>69 years) increased by 76%. The observed survival rates correlated with tumour stages. The overall observed 5-year survival rate (stage I-IV) was 51 ± 3% (95% confidence interval).

**Conclusion:**

The increasing incidence rates of colon adenocarcinomas, the persistence of advanced tumour stages (stage III), the mortality rates which remain stable, and the changing trends in the age- and sub-site distribution underline the need for preventive measures at the age of 50 in asymptomatic patients to reduce mortality from colo(rectal) cancer.

## Background

Since 1994, colorectal adenocarcinoma has been the third most frequent cancer in the Grand-Duchy of Luxembourg (Western Europe), preceded by breast and prostatic cancer [1-3]. In the United States of America (USA), Japan and France, there have been changes in the site distribution of large bowel cancers over the last decades [4-9]. Because of the lack of a systematic, organized screening for colo-rectal cancer in Luxembourg, the data presented here may help to define new preventive screening approaches, to increase funding and hasten the introduction of screening for this tumour. The aim of this study was to investigate changes in incidence, stage, anatomical site distribution, and the observed survival rates of colon cancer over two 5-year periods, 1988–1992 and 1994–1998. Analogous to the report on rectal cancer published earlier [10], descriptive epidemiological data on colon cancer were collected to underline the need for the initiation of a nation-wide strategy for the early detection of colorectal cancer.

## Methods

Between January 1^st^, 1988 and December 31, 1998, all consecutive new cases of high-grade adenoma and all new cases of invasive colonic adenocarcinoma (COAC) were registered by the national Morphologic Tumour Registry (MTR) in Luxembourg. These cases had been exclusively diagnosed and "double-read" in the central department of pathology by nine senior pathologists. Over the decade the population increased from 374,900 to 429,000, – an average increase of 1.3% per year. In the elderly age group >69 years of age, there was a continuous increase by 1.8% (0.5 – 4.4%) [11]. Patients of all nationalities, race or ethnical origin living in Luxembourg were considered. Disease histology was limited to high-grade adenomas (i.e. adenomas with severe dysplasia) and invasive adenocarcinomas of the colon. Tumours recorded as other carcinomas, metastatic or recurrent disease, metachronous adenomas or invasive adenocarcinomas, malignant carcinoid and mesenchymal malignant tumours were excluded from the current review. For patients with synchronous colon neoplasias, only cases with the worst stage category were taken in account.

Data on patients with high-grade adenomas (i.e. stage 0 patients), who by definition could not have metastases in regional lymph nodes or distant metastases, were obtained from 254 biopsy-forceps and polypectomy specimens removed by 26 gastroenterologists and 11 senior-surgeons. Adenomatous proliferations in marginal zones of invasive adenocarcinomas and adenomas with severe dysplasia appearing as recurrent invasive disease within 2 years at the same site were not taken into account.

Data on 1158 surgical resection specimens allowed an interpretation in relation to the local and regional spread, the lymph node and the residual tumour status. Hence the stratification of the invasive colonic adenocarcinomas by stage (stage I-IV) led to the exclusion of patients with biopsy diagnosis alone and patients who had only undergone polypectomy without segmental resection (i.e. without regional lymphadenectomy). Patients with known preoperative debulking radiotherapy were not considered. The surgical resection specimens were analysed with respect to tumour-stages (stage I to IV), according to the guidelines of the Union Internationale Contre le Cancer (UICC) and the American Joint Committee on Cancer (AJCC) [12-14]. We defined stage I and stage II as early cancers and stage III and stage IV as advanced colon cancers.

To evaluate the influence of the number of colonoscopic procedures on the incidence rates and the stage distribution of colonic cancers in Luxembourg, the data on colonoscopies (1992–1998), gratefully made available by the National Health Found (NHF), were analysed [15]. The R-classification according to the TNM-system was performed by conventional methods [16,17]. In our series the histological examination of the proximal, distal and lateral (deep) line of resection was mandatory. R0 indicates no residual tumour; R1 corresponds to microscopically and R2 to macroscopically residual tumour, while Rx did not allow a statement on residual tumour.

The histopathological diagnoses were made according to the WHO-classification [18]. On this basis, we defined 'high grade' – adenomas, the equivalent of stage 0 or former adenocarcinoma in situ, as adenomas of tubular, villous or tubulo-villous histological type with high-grade intraepithelial neoplasia (i.e. severe dysplasia). This category includes cancer cells confined within the glandular basement membrane (intraepithelial) or lamina propria (intramucosal) with no extension through the muscularis mucosae into the submucosa.

We considered all colon segments as large bowel, with the exception of the rectum and the recto-sigmoid junction. Anatomically the rectum was defined as a 16 cm long segment between the ano-cutaneous line and the sigmoid colon. Cancers described at the recto-sigmoidal junction without statement on the centimetres from the anal margin were considered as rectal adenocarcinomas and were excluded. Left-sided colon cancers were defined as adenocarcinomas of the descending colon and the sigmoid colon. Right-sided colon cancers included adenocarcinomas situated above the splenic flexure (i.e. splenic flexure, transverse colon, hepatic flexure, ascending colon and caecum with its prolongation, the appendix vermicularis).

Beside the evaluation of the frequency of invasive COCA by the 17 'classic' 5-year age categories (0–4 years to 80 years and above) the changes of anatomic sub-site distribution in relation to 3 age groups young (<40 years of age), middle aged (40–69 years) and elderly (70 years and above) patients were analyzed. The observed survival rates of patients with colon cancer were measured from the time of diagnosis and calculated by the actuarial method (life-table) with a 95% confidence interval (c.i.). To evaluate the observed survival rates in relation to the anatomical extent (stage I-IV) and the surgical resection margins (R-status), 164 patients with colon adenocarcinoma diagnosed only by biopsy-forceps without consecutive surgical resection data in our files, who were without final stage and who are part of the 1379 patients with an invasive COCA, were analysed separately.

The mortality rates of colon cancer were issued from the anonymous files of the death certificates published by the National Health Direction (Ministry of Health) [19]. The age-standardized incidence rates of the colon cancers diagnosed in the Grand-Duchy of Luxembourg during the period 1983–1997 were compared with the data of other geographical European regions with similar population density and socio-economic characteristics, published by the WHO in "Cancer in Five Continents, volumes VI, VII, VIII" [1-3,20-22].

The statistical evaluations include the chi-square test (χ^2^) with a level of significance p < 0.05 and the life-table survival analysis. The age-standardized incidence rates were calculated by the direct method, the standard error of the age-standardized rates by the Poisson approximation [23].

## Results

The histopathological diagnoses of 1379 consecutive invasive colon adenocarcinomas (COAC) were provided from 164 samples obtained by biopsy only (11.9%), 57 polypectomy specimens (4.1%) and 1158 surgical resection specimens (84.0%). The latter allowed a stratification by stage (UICC/AJCC, 1997) and had reliable follow-up data. The 1379 invasive adenocarcinomas of the colon involved 658 males (47.7%) and 721 females (52.3%); M/F-ratio of 1: 1.1. The mean age was 69.7 years (range: 26–97). During the study period, 254 patients were diagnosed with high-grade adenomas. These patients constitute a separate group from the 1379 invasive COAC.

**Figure 1:**
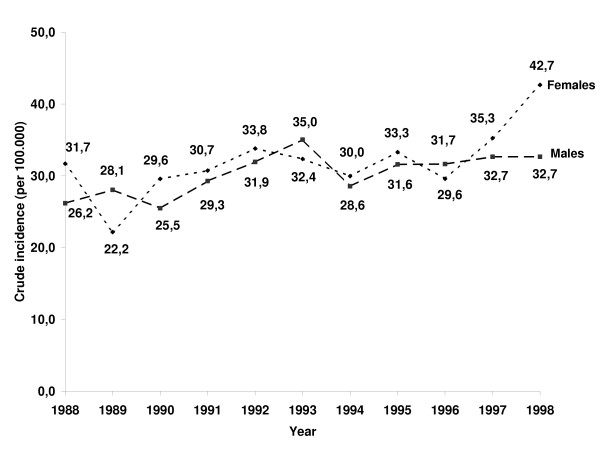
Time trend of the crude incidence rates of the invasive colon adenocarcinomas (n = 1379) by gender; 1988–1998.

The average, annual, age-specific rates of colon AC in Luxembourg over the period 1988–1998 were 31.1 per 100,000 for all ages and both genders, for males 30.3 per 100,000 and for females 31.9 per 100,000 (Figure 1). The comparison of the crude incidence rates of patients with colon AC diagnosed in the 5-year periods 1988–1992 and 1994–1998 revealed an increase by 13.5% for both genders from 28.9 to 32.8, for females by 15.5% from 29.6 to 34.2 and for males by 11.3% from 28.2 to 31.4 per 100,000.

The average, annual, age-standardized incidence rates (ASR-world population) of colon cancer (period 1988–1998) were 17.5 per 100,000, for males 20.5 per 100,000 and for females 15.4 per 100,000. Table 1 documents the world age-standardized incidence rates (ASR/W) of the invasive COAC in the Luxembourgish population (females and males) over the period 1983–1997 in comparison to other European countries.

The time trend (1988–1998) of the frequency of invasive COAC showed that the total number of invasive COAC rose significantly from 109 cases in the year 1988 to 162 cases in 1998 (in females from 61 to 93, in males from 48 to 69). Comparing the two 5-year periods 1988–1992 and 1994–1998, there was a significant increase in the absolute number of all COAC from 557 to 687 cases (p < 0.001). The absolute number of COAC in males increased significantly from 266 to 323 cases (p < 0.02), and in females significantly from 291 to 364 cases (p < 0.01).

**Figure 2:**
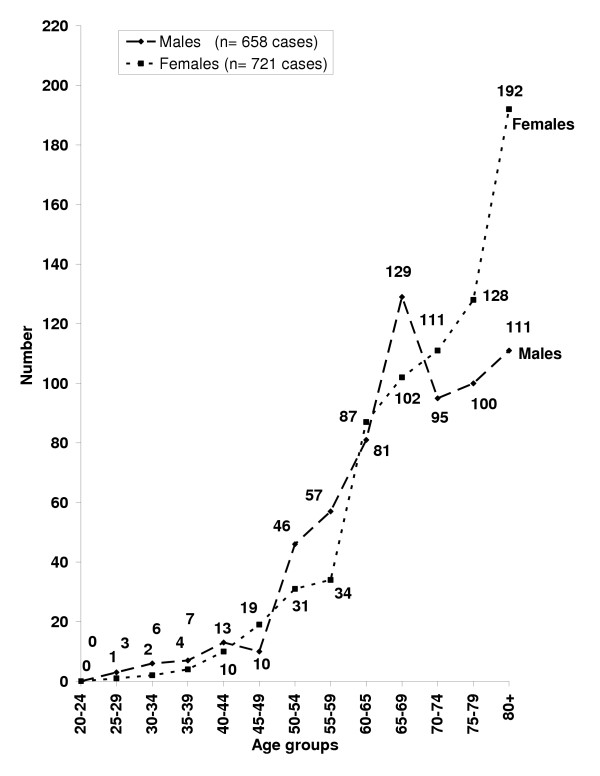
Age-distribution of the invasive colonic adenocarcinomas (n = 1379 cases); 1988–1998.

Figure 2 shows the age-distribution of all patients with histologically confirmed invasive colonic adenocarcinoma (n = 1379 cases). Of these patients 1.7% were <40 years of age; 3.8% between 40 and 49 years; 41.0% between 50 and 69 years; 31.5% between 70 and 79 years and 22.0% of the patients were 80 years and above.

Concerning the COAC stage distribution, two peculiarities appeared at the extremes of patient age scales. Below the age of 40, none of the patients (n = 23) had early stage cancer (i.e. stage I or stage II). On the other hand, out of the 303 patients aged 80 years and above, most had stage II (39.0 %) and stage III (38.2 %) disease, whereas stage I (10.6 %) and stage IV (8.3 %) were much less frequent.

**Figure 3:**
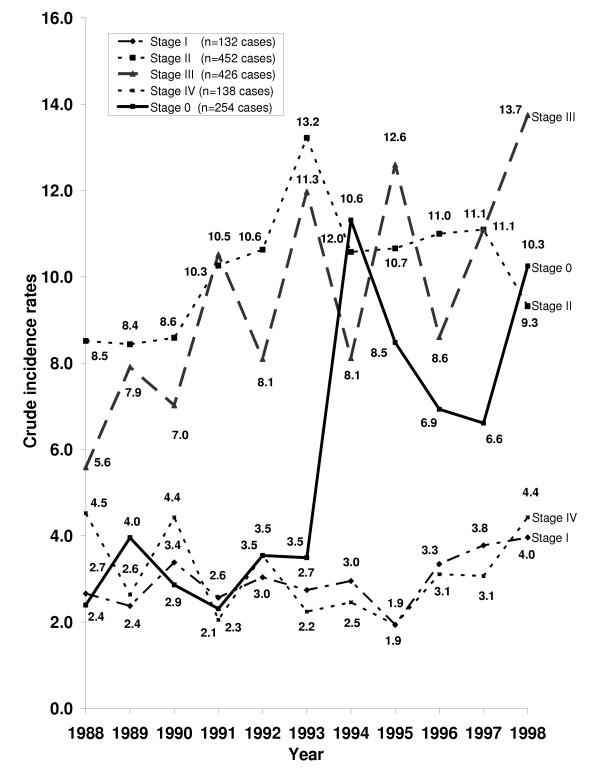
Crude incidence rates of the high-grade adenomas (n = 254) and the invasive adenocarcinomas of the colon (n = 1379) stratified by stage (TNM/UICC-AJCC, 1997) [14].

Time trends in relation to the tumour-stages [TNM-System (UICC/AJCC] are represented in Figure 3. During the two 5-year periods 1988–1992 and 1994–1998, there was a highly significant increase (p < 0.001) in the crude incidence rates of the stage 0 cases (i.e. high-grade colonic adenomas) by 190%, an increase in stage I cases by 14.3%, in stage II cases by 12.9%, in stage III cases by 38.5%, and a decrease in stage IV cases by 11.8%.

**Figure 4:**
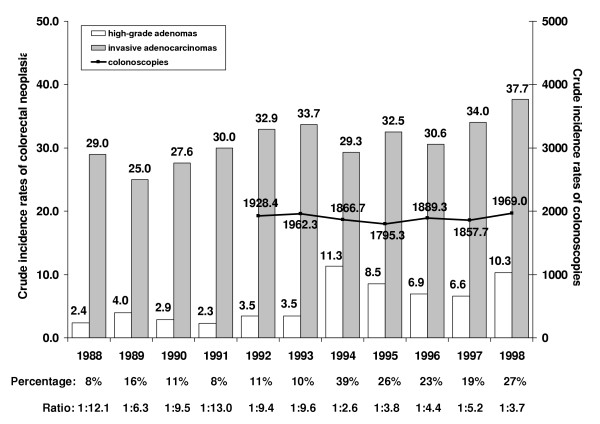
Crude incidence rates and colonic high-grade adenoma / invasive adenocarcinoma-ratio in comparison to colonoscopies: 1988–1998; both genders; n = 254*/1379 cases (*diagnoses by biopsy only included).

The relation of the 254 new colonic high-grade adenomas to the 1379 new invasive adenocarcinomas stratified by years is shown in Figure 4. The comparison of the average of crude incidences of the high-grade adenomas to the invasive adenocarcinomas for the two 5-year periods indicates a highly significant improvement of the ratio by 148% in the second period. There were no significant changes in the number or crude incidences of colorectal endoscopies or colonoscopies with biopsy examinations.

**Figure 5:**
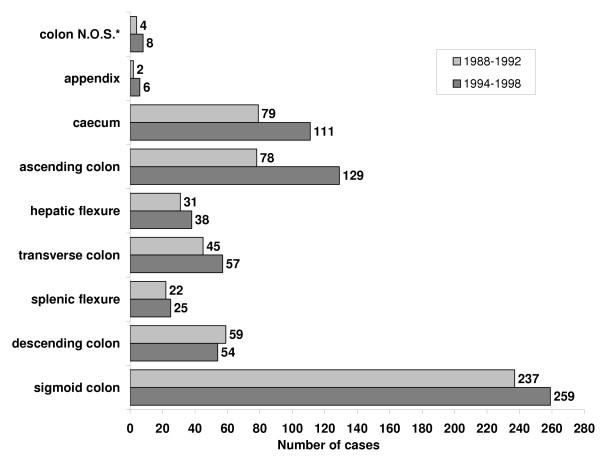
Number of invasive colonic adenocarcinomas (n = 1244; cases of the year 1993 excluded) by anatomic site; 1988–1992 and 1994–1998. (N.O.S.* = not otherwise specified)

The distribution of the 1244 new invasive colon adenocarcinomas by anatomical site and two 5-year periods (1988–1992: n = 557 cases and 1994–1998: n = 687 cases) are given in Figure 5. Twelve cases (1.0%), which were not otherwise specified, had to be excluded. In relation to three age groups (< 40 years of age, 40 – 69 years and >69 years) the anatomical sub-site distribution shows a shift to right-sided colon adenocarcinomas by 9.2% in the 40 to 69 years age group (n = 553) and by 76.2% in the group of patients of >70 years of age (n = 658). The number of the left-sided colon cancers decreased by 14.5% in the 40–69 years age group and increased by 22.9% in those >70 years. Patients with no precise site and those of the year 1993 were excluded for statistical (two 5-year periods) comparison reasons. In Table 2 the anatomical sub-site distribution in relation to the three age groups and their age-specific rates per 100,000 is represented.

The overall observed survival rates of the 1379 patients with an invasive COAC, diagnosed by biopsy or polypectomy or surgical resection specimen were calculated and stratified by years: 1-year: 72 ± 2% (n = 997/1379); 2-year: 60 ± 3% (833/1379); 3-year: 54 ± 3% (n = 740/1379); 4-year: 48 ± 3% (n = 662/1379) and 5-year 44 ± 3% (n = 604/1379).

In Table 3, the observed 5-year survival rates of 931 invasive COAC stratified by stage and operation for cure (R0), and those of 58 patients with COAC with microscopically (R1) or macroscopically (R2) residual tumour after surgical resection are represented. The analysis of the observed survival rates of the 164 patients with colon AC, diagnosed by biopsy-forceps only (i.e. without known final stage), revealed that 70 patients (42.7%) survived 12 months, whereas 20 (12.2%) died in the first month after diagnosis, 46 (28.1%) within the next 6 months and 28 (17.1%) in the period from 6 to 12 months. The age-distribution of these patients showed that 10 (6.1%) patients were <50 years, 105 (64.0%) were aged between 50 and 79 years and 49 (29.9%) were >80 years of age.

The mortality rates for colon cancer in the Grand-Duchy of Luxembourg over the last 15 years (1984–1998), grouped by 5-year periods, did not change significantly (Table 4) [19]. There was a slight, insignificant (p < 0.05) decrease in the crude death rates from 26.3 per 100,000 in the period 1984–1988 to 23.8 per 100,000 in the period 1989–1993 and 23.9 per 100,00 in 1994–1998 [19].

## Discussion

In contrast to the USA and Canada, the age-standardized incidence rates of colon cancer in Luxembourg steadly increased from 1983–1997 for both genders (Table 1). Our findings are in concordance with those of many other European countries, where the increase occurs mainly in the male population [20-22,24]. Some authors believe that the decreasing incidence rates in the USA are due to preventive cancer screening [25]. We found 3.2% cases with synchronous colon adenocarcinomas, which is comparable to the 3.0% and 4.8% of synchronous cancers identified by authors in other countries [26,27].

The effect of age was examined in relation to the classic age groups and in relation to three cohorts of patients, (a) under 40 years of age, (b) between 40 and 69 years, and (c) 70 years and older. The number of colon cancers increases around the age of 45 (Figure 2). This is consistent with the findings of Imperiale et al., who found a low incidence of colon adenomas in the age groups 40 to 49 years [28]. Patients under 40 years of age had a higher incidence of advanced stage colon cancers. Similar observations were reported by Mostafa et al. [29].

Considering that high-grade adenomas are obligate precancerous lesions with a slow progression, that polypectomy can mean prevention of cancer, and that the high-grade adenoma/invasive adenocarcinoma ratio (Figure 4) improved in the late 1990's, we observed evidence of earlier diagnoses [30]. We consider the continous registration of high-grade adenomas and determination of the adenoma-adenocarcinoma ratio as a useful additional quality assurance marker in evaluating clinical and histopathological diagnostic procedures. To explain the unexpected increase of precancerous colonic lesions, we analysed our files in order to identify parameters that might have changed, such as staff composition or other factors. Over the period 1992–1995 the number of physicians (n = 38) practicing endoscopy did not change and the number of high-grade adenomas (n = 109 cases) removed by each endoscopist varied between one and six cases. Considering that, the number and the crude annual incidence rate of the colonoscopies did not rise significantly over the time, that the staff of pathologists remained the same, the histopathological criteria and coding did not change, and that a systematic organized screening programme until the year 2000 was not launched and a spontanous, unorganized screening is not known, we believe that a more selective indication for colonoscopy examinations may be one of the causes of this increase. The abrupt change after 1993 in the number of stage 0-cases may be the consequence of the changes in the statute of the National Health Fund. In 1992 the notions of patients and medical care providers 'profiling' at the national level were introduced. At the same time, the media campaign for preventive medical care strategies against cancer (breast, malignant melanoma) launched by the governmental institutions in collaboration with the NHF and the Luxembourg Foundation against Cancer may have had a beneficial (secondary) effect. Not only the incidence of stage 0-cases rose, but we found also a positive influence on the crude incidence rates of stage I and stage II patients increasing over the two 5-year period (1988–1992) and (1994–1998) by 14.3 and 12.9% respectively. Unfortunately there has been a highly significant (p < 0.001) increase in stage III patients by 38.5% too. Colon AC in young patients (40 years of age or less) seems to be more aggressive, since those patients were diagnosed exclusively in stage III and stage IV. Since these patients often present a positive family history of colorectal cancer, there is an obvious need for health-care providers to heighten awareness, and to identify high risk persons in this population group [25,31,32].

In North America, Japan, France and other high-risk areas, there has been a proximal shift in the sub-site distribution of colorectal cancer [4,29,32-34]. Similar changes have occurred in Luxembourg. This proximal shift was found in both sexes, especially in patients aged 70 and above. In males the increase was 14.0%, in females 73.6%. Mitry et al. [33] found that right colon cancer rates had increased by 21.6% in males and 10.4% in females. This underlines the need to discuss the opportunity for total colonoscopy in patients of higher risk or elderly age groups [30].

In our cohort, the overall 5-year survival rates of the patients with surgery for colon cancer without distinction of the residual tumour-status were 46 ± 3% (95% c.i.). Because the data on age-specific risk of death from other causes than colon cancer during the 1990's are not yet available, the relative survival rates could not be calculated. It must be admitted that relative survival rates will give better data for colon cancer in Luxembourg than indicated by the observed survival rates reported here. The relative 5-year survival rates reported in the literature vary between 30.6 and 55.3% [6,35].

Because COAC incidence increases at the age 50 approximately in our series, because a significant shift to right-sided colon cancer has been detected in patients of 70 years of age and above and because survival rates would probably improve if the disease is treated in its early stages, a strategy to perform twice-lifetime colonoscopy (at ages 50 and 60 years or at the transition to the pension age with 65 years) for asymptomatic patients without family history or other risk factors, as discussed by others, needs further investigations [4,29,34,36,37]

## Conclusion

In contrast to the time trends in the USA and Canada, we found increasing age-standardized incidence rates in colonic adenocarcinomas and an increase of the crude incidence rates especially in stage III patients. Despite the 4-fold increase in high-grade adenoma diagnoses (i.e. stage 0 cases), efforts should be reinforced in preventive strategies to detect more precancerous lesions and/or early tumour-stages, to reduce the true incidence of advanced tumour-stages at diagnosis, and by this the mortality rates. The findings of this study should provide baseline information and scientific evidence for the elaboration and implementation of cancer preventive and intervention strategies to those populations at average and high-risk for colorectal cancers. As long as new diagnostic procedures, such as stool-based DNA-test for colorectal tumours, are not yet available for mass-screening purposes, and as long as the effectiveness of a complete colonoscopy screening for colorectal cancer in asymptomatic patients beginning at the age of 50 has not been proven by population-based controlled trials, the data underline the need of at least a systematic faecal occult blood test (FOBT) screening for colorectal cancer beginning at the age of 50 [30,38,39]. Our data support mainly that screening could be considered in preventing deaths from colo(rectal) cancer. The findings can be used by the national health care authorities to define a screening strategy and to help in deciding the target age-groups in Luxembourg.

## Competing interests

The author(s) declare that they have no competing interests.

## Authors' contributions

All authors (RS, PP, YW, NK, CC) collaborated intensely on all aspects of the manuscript, from research design to data preparation and presentation. RS wrote, and all authors approved the final manuscript.

## Pre-publication history

The pre-publication history for this paper can be accessed here:



## Figures and Tables

**Table 1:** Colon cancer in the European Community: world age-standardizedincidence rates [ASR(W)*], time trends: 1983–1987; 1988–1992; 1993–1997.

**Males**	**83–87**	**88–92**	**93–97**		**Females**	**83–87**	**88–92**	**93–97**	
F/Bas Rhin	27.2	30.2	31.3	↑	D/Saarland	19.4	20.4	21.5	↑
D/Saarland	23.7	25.5	27.3	↑	NL/Maastricht	17.1	17.9	19.4	↑
I/Parma	23.0	24.4	27.0	↑	DK/Denmark	19.3	19.9	18.6	↓
IRL/Southern	21.8	24.2	25.3	↑	IRL/Southern	19.7	20.3	18.3	↓
E/Navarra	13.8	19.2	22.1	↑	I/Parma	16.1	17.8	17.9	↑
NL/Maastricht	19.7	21.9	22.1	↑	UK/South Western	15.9	16.2	17.3	↑
UK/South Western	18.2	19.2	22.1	↑	F/Bas Rhin	17.4	18.8	16.9	↓
**L/Luxembourg**	15.5	20.2	**21.9**	↑	**L/Luxembourg**	11.3	14.9	**15.0**	↑
DK/Denmark	20.3	20.6	20.7	↑	S/Sweden	15.8	15.9	15.0	↓
CH/St.Gall	16.4	16.5	19.7	↑	E/Navarra	9.6	13.0	12.4	↑
S/Sweden	17.5	17.7	17.7	↔	CH/St.Gall	11.7	12.0	12.1	↑
SF/Finland	11.9	12.8	14.5	↑	SF/Finland	10.6	11.6	12.1	↑
									
CND/Canada	27.8	26.9	25.9	↓	CND/Canada	23.6	21.3	20.0	↓
USA/Seer**, white	31.1	28.1	25.5	↓	USA/Seer**, white	23.6	20.8	19.4	↓

* Cancer incidence in five continents. Vol VI; Vol VII; Vol VIII [20, 21, 22]

** SEER: Surveillance, Epidemiology and End Results Program

**Table 2:** Invasive colon adenocarcinomas (n = 1232*) by anatomic site (left and right colon), by age groups (<40 years; 40–69 years; >69 years) and age-specific incidence rates; 1988–1992 and 1994–1998.

**Age groups**	**Number of incident cases (ri)**	**Person- years of obervation (ni)**	**Age-specific rates per 100,000 (ri/ni)**	p-values **
**<40 years**				
colon right 1988–1992	5	1 072 099	0.5	p = n.s.
colon right 1994–1998	9	1 151 878	0.8	
colon left 1988–1992	2	1072 099	0.2	p = n.s.
colon left 1994–1998	5	1151 878	0.4	
**40–69 years**				
colon right 1988–1992	130	676 772	19.2	p = n.s.
colon right 1994–1998	142	742 577	19.1	
colon left 1988–1992	150	676 772	22.2	p = n.s.
colon left 1994–1998	131	742 577	17.6	
**>69 years**				
colon right 1988–1992	122	175 629	69.5	p < 0.001
colon right 1994–1998	215	196 145	109.6	
colon left 1988–1992	144	175 629	82.0	p = n.s.
colon left 1994–1998	177	196 145	90.2	

* excluded colon cancers N.O.S.(n = 12) and those of 1993 (n = 135)

** level of significance p < 0.05; n.s. = not significant

**Table 3:** Prognoses of invasive colonic adenocarcinomas resected for cure (R0) or with residual tumour (R1+R2) and stratified by stage-TNM/UICC [12, 13, 14, 23].

**Stage**	**TNM**	**Observed 5-year survival rates (actuarial method, 95% confidence interval)**		
**UICC, 5^th ^edition**		**R0**	**R1+R2**	p-values ***

I *	T1 N0 M0 T2 N0 M0	71 +/- 8% (n = 129)	100% ** (n = 1)	<0.5188
II *	T3 N0 M0 T4 N0 M0	63 +/- 5% (n = 388)	45 +/- 30% (n = 11)	<0.2392
III *	anyT N1 M0 anyT N2 M0	38 +/- 5% (n = 331)	4% ** (n = 25)	<0.0007
IV *	anyT anyN M1	17 +/- 8% (n = 83)	5% ** (n = 21)	<0.1584

I-IV *		51 +/- 3% (n = 931)	14 +/- 9% (n = 58)	<0.0001

* only surgical specimens with available survival data

** number of cases too low for calculating the 95% confidence interval

*** level of significance p < 0.05

**Table 4:** Analysis of the mortality rates from all colon adenocarcinomas of the last 15 years (1984–1998) declared in Luxembourg by death certificate only [19].

	**Males**	**Females**	**both genders**	p-values*
**1984–1988:**	26.5/10^5^	26.2/10^5^	26.3/10^5^	
				p = n.s.
**1989–1993:**	22.5/10^5^	25.0/10^5^	23.8/10^5^	
				p = n.s.
**1994–1998:**	23.6/10^5^	24.3/10^5^	23.9/10^5^	

* level of significance p < 0.05 ; n.s. = not significant for both genders
